# Computed Tomography and Magnetic Resonance Imaging Findings in Early Glioblastoma (e-GBM): Spotting the Wolf in Sheep’s Clothing

**DOI:** 10.7759/cureus.92895

**Published:** 2025-09-22

**Authors:** Aiyapa A Ajjikuttira, Sarah Li, Mohammadreza Haghighatpanah, Pranav Sharma, Rosalind Jeffree, Jennie Roberts

**Affiliations:** 1 Department of Medical Imaging, Royal Brisbane and Women's Hospital, Brisbane, AUS; 2 Department of Radiation Oncology, Royal Brisbane and Women's Hospital, Brisbane, AUS; 3 Department of Neurological Surgery, Royal Brisbane and Women's Hospital, Brisbane, AUS

**Keywords:** computed tomography (ct ), early glioblastoma, glioblastoma gbm, glioblastoma radiology, magnetic resonance imaging (mri)

## Abstract

Introduction: Radiological differences exist between early-stage and late-stage glioblastoma (GBM). Most clinicians are aware of the latter appearances, but in our experience, early GBM (e-GBM) is often missed due to the absence of these classical findings. In this retrospective cohort study, we aim to highlight the radiological findings of e-GBM in order to improve recognition. This is important, as earlier recognition and treatment of this serious condition may improve prognosis.

Methods: A retrospective study of all patients presented at our institutional neuro-oncology multidisciplinary team meeting between 2012 and 2023 was undertaken. All patients had histologically confirmed GBM and early imaging that did not demonstrate the typical characteristics of malignant tumour. Imaging performed prior to the development of classical magnetic resonance imaging (MRI) features was reviewed by a single experienced neuroradiologist to investigate common imaging characteristics of e-GBM.

Results: Thirty-four patients (21 male, 13 female) were included. All patients presented with neurological symptoms and underwent MRI, with 30 of 34 patients having gadolinium-enhanced MRI. On T2-weighted and fluid-attenuated inversion recovery (FLAIR) sequences, all 34 patients had cortical signal change, while 33 of 34 patients had signal change in the subcortical region. All regions of FLAIR and T2 signal change demonstrated associated restricted diffusion. Thirty-two of 34 patients underwent computed tomography (CT) prior to MRI. Twenty-three of 32 patients had hyperdensity on CT correlating with areas of signal change on MRI.

Conclusion: e-GBM should be considered in patients presenting with neurological symptoms, in particular seizures, when T2WI or FLAIR signal abnormalities in a cortical or subcortical location are seen with associated restricted diffusion. Hyperdensity on non-contrast CT imaging congruent with areas of signal change on MRI should also prompt clinicians to consider e-GBM as a differential diagnosis.

## Introduction

Glioblastoma (GBM) is the most common and aggressive adult primary cerebral malignancy. Currently, fewer than five percent of all GBM patients survive beyond five years despite optimal management [1].

The 2021 World Health Organisation (WHO) Classification of Tumours of the Central Nervous System defines GBM as an isocitrate dehydrogenase (IDH)-wildtype diffuse astrocytic glioma. Other key criteria include the presence of necrosis or microvascular proliferation on histological examination, or one of three molecular features: TERT promoter mutation, EGFR amplification, and combined gain of chromosome seven and loss of chromosome 10 [2]. IDH-mutated GBMs have been reclassified as astrocytomas in the updated WHO classification system [3].

Early imaging diagnosis, and therefore early treatment, has the potential to improve patient outcomes. Aggressive resection is associated with improved overall survival in newly diagnosed patients [4], while large GBMs correlate with poorer prognosis and are less likely to be completely resected [5]. Due to the rapid growth of GBMs, it is unusual for radiologists to encounter them early [6], and therefore, the characteristics of the early stages of tumour growth are not widely known or recognised. This paper is designed to educate the reader on these features of magnetic resonance imaging (MRI) and computed tomography (CT).

Gadolinium-enhanced MRI is the preferred imaging modality for intracranial neoplasms, including GBM [7]. The histopathological hallmarks of GBM include tumour infiltration, hypoxia, angiogenesis, and necrosis. These features account for the classic and well-described appearances of established GBM seen on imaging, namely irregular contrast enhancement, central necrosis, mass effect, and perilesional oedema [8].

It is postulated, however, that in the pre-clinical phase, not all these characteristic imaging features exist. These so-called “early-stage” GBM (e-GBM) are often misdiagnosed on imaging as other pathologies, including acute infarct (especially with restricted diffusion) [8], low-grade glioma [9], metabolic disease [9], cortical laminar necrosis, encephalitis [8,10], and demyelinating lesions.

We undertook a review of the literature surrounding the imaging features of e-GBM and conducted a retrospective cohort study of e-GBM patients from our institution. We hope to highlight important imaging features that should prompt clinicians to consider e-GBM as a differential diagnosis.

## Materials and methods

Ethical approval was granted by our institutional review board for this retrospective cohort study (LNR/2018/QRBW/45566). A waiver of patient consent was obtained as part of the ethics approval.

Literature review

We undertook a review of the literature, searching the PubMed, Embase, and SCOPUS databases for articles discussing MRI findings in e-GBM. Articles authored in English that were published prior to May 2023 were included. All identified literature underwent title and abstract screening and proceeded to full-text screening if they addressed imaging findings on MRI that showed e-GBM.

All relevant studies examined in our work were retrospective cohort studies [6,8,9,11-18]. While case reports were identified in our literature search, these were excluded as they had previously been discussed elsewhere [9]. A total of 11 articles were identified through our search, with all search strings presented in tabular format in the appendix.

Observational cohort study

We performed a retrospective study of all patients who were presented at our institutional neuro-oncology multidisciplinary team meeting between 2012 and 2023 with histologically confirmed GBM and earlier imaging that was not recognised to show aggressive malignant tumour.

The cases were identified by an experienced neurosurgeon (RJ) and neuroradiologist (JR) from multiple centres in Queensland, Australia. CT and MRI imaging were then reviewed by a neuroradiologist (JR). All cases had to have histopathological confirmation of being GBMs. Cases were excluded if their imaging revealed typical MRI findings of GBM, as detailed above. Details of the clinical presentation were obtained from the initial request form for the imaging.

A total of 34 patients met the inclusion criteria. Patient age, sex, clinical presentation, and the provisional diagnosis offered on MRI by the initial reporting radiologist were documented. The early imaging, before the development of characteristic peripheral enhancement and central necrosis, was reviewed by a single experienced neuroradiologist (JR) to determine common diagnostic characteristics of e-GBM. Imaging findings across T1WI, T2WI, FLAIR, DWI, and ADC sequences were examined. Due to the small sample size (n = 34), only descriptive statistics were reported, without inferential analysis. Data were analysed using Microsoft Excel, as part of the Office 2024 Professional Plus suite (Microsoft Corporation, Redmond, Washington). Table 1 succinctly outlines the pertinent imaging features in our patient cohort.

**Table 1:** Imaging features encountered in our patient cohort. CT: computed tomography, MRI: magnetic resonance imaging.

Imaging feature	Number of patients	Percentage (rounded to nearest whole)
Contrast-enhanced MRI	30	88%
Non-contrast MRI	4	12%
Non-cortical/non-subcortical signal change	12	35%
Presence of restricted diffusion on imaging review	33	97%
Corresponding findings on CT preceding MRI (n=32)	29	90%

## Results

Our series, one of the largest examining e-GBM patients, comprised 34 patients (21 male, 13 female). The median age at the time of diagnosis was 61 years (53-68 years; IQR 15 years). The median survival in our series was 17 months (10-29 months; IQR 19 months). Eighteen patients had primary left-sided lesions, and 16 patients had primary right-sided lesions. All patients presented with neurological changes, including seizure, visual disturbances, weakness, facial droop, and expressive dysphasia.

All patients had T2WI (T2 weighted imaging), FLAIR (fluid-attenuated inversion recovery), ADC (apparent diffusion coefficient), and DWI (diffusion weighted imaging) sequences. Thirty of 34 patients (88%) had T1WI sequences with gadolinium, consisting of either turbo spin echo (TSE), magnetisation-prepared rapid acquisition gradient echo (MPRAGE), and/or T1 fat saturation (T1 fat sat) sequences. Table 2 provides a comprehensive overview of lesion characteristics and imaging appearances.

**Table 2:** Patient demographics and imaging details. ADC: apparent diffusion coefficient, ATOR: at time of review, DWI: diffusion weighted imaging, FAT SAT: fat saturation, FLAIR: fluid attenuated inversion recovery, HGG: high-grade glioma, LGG: low-grade glioma, MPRAGE: magnetization-prepared rapid acquisition gradient echo, PDx: provisional diagnosis. PRES: posterior reversible encephalopathy syndrome, SPACE: sampling perfection with application optimized contrasts using different flip angle evolutions, T1WI: T1 weighted imaging, T2WI: T2 weighted imaging, TSE: turbo spin echo.

Patient number	Age and sex	Presentation	Lesion location	Survival	FLAIR and T2WI		T1WI post-contrast		Restricted diffusion		PDx on MRI	CT findings
					Cortical or subcortical signal change	Signal change elsewhere	T1 sequence	Presence of enhancement	Reported at initial read	Present on review		
1	72 years 3 months, M	Seizure	Right hippocampus	5 months	Present	Left medial temporal lobe	No contrast given	No contrast given	Reported as present	Present	Post-seizure, limbic encephalitis, LGG.	No comment ATOR. Hyperdense retrospectively.
2	49 years 6 months, M	Seizure	Right temporo-occipital junction	30 months	Present	None	TSE	Present	Reported as present	Present	High-grade glioma	Normal ATOR. Normal retrospectively.
							MPRAGE	Present				
3	73 years 4 months, F	Decreasing memory and ataxia	Left-sided thalamus, medial temporal lobe, claustrum	2 months	Present	Right temporal lobe	TSE	Present	Reported as present	Present	Autoimmune vs neoplastic processes	Hypodense ATOR. Mixed hyper and hypodensity retrospectively.
							MPRAGE	Present				
4	58 years 6 months, M	Seizure	Left superior temporal gyrus	30 months	Present	None	MPRAGE	Present	Reported as not present	Present	Postictal vs glial tumour	Normal ATOR. Hyperdense retrospectively.
5	68 years 5 months, M	Seizure	Right superior frontal gyrus	1 month	Present	None	MPRAGE	Present	Reported as present	Present	HGG	Normal ATOR. Hyperdense retrospectively.
6	38 years 11 months, M	Focal seizure	Right paracentral lobule	31 months	Present	Right basal ganglia	MPRAGE	Not Present	Reported as present	Present	LGG with associated post-ictal changes	Normal ATOR. Hyperdense retrospectively.
							T1 SPACE FAT SAT	Present				
7	60 years 1 month, F	Stroke-like symptoms	Left occipital	Alive (10 months)	Present	None	MPRAGE	Not present	Reported as not present	Not present	Indeterminate lesion	Loss of grey-white matter differentiation ATOR. Hypodense retrospectively.
8	67 years 6 months, M	Dysarthria	Left operculum	11 months	Present	None	MPRAGE	Present	Reported as present	Present	Indeterminate lesion	Hyperdense ATOR. Hyperdense retrospectively.
9	60 years 2 months, M	Seizure	Right frontal lobe	24 months	Present	Left temporal stem	Present	Present	Reported as not present	Present	Reported as normal	Normal ATOR. Hyperdense retrospectively.
10	42 years 10 months, M	Seizure	Right cingulate gyrus	14 months	Present	None	MPRAGE	Not present	Reported as present	Present	Subacute infarct, seizure-related oedema, glioma.	Hyperdense ATOR. Hyperdense retrospectively.
11	39 years 9 months, M	Seizure	Left temporal lobe	14 months	Present	None	MPRAGE	Present	Not reported	Present	Herpes encephalitis, LGG	Normal ATOR. Hyperdense retrospectively.
12	51 years 6 months, M	Seizure	Left superior frontal gyrus	15 months	Present	None	TSE	Present	Reported as present	Present	Lymphoma, metastatic malignancy.	Normal ATOR. Hyperdense retrospectively.
13	64 years 6 months, F	Left-sided headache and dysarthria	Left temporal lobe	20 months	Present	None	MPRAGE	Not present	Reported as present	Present	Low-grade intermediate glioma	Normal ATOR. Hyperdense retrospectively.
14	56 years 11 months, F	Seizure	Right precentral gyrus	17 months	Present	None	MPRAGE	Present	Not reported	Present	LGG	Normal ATOR. Hyperdense retrospectively.
15	66 years 8 months, M	Acute dysphasia	Left temporal lobe	17 months	Present	None	MPRAGE	Present, smudgy	Reported as present	Present	Infarct, early GBM.	Normal ATOR. Hyperdense retrospectively.
16	64 years 2 months, M	Seizure	Right temporal lobe	29 months	Present	Right pulvinar	MPRAGE	Present	Reported as present	Present	Encephalitis, autoimmune, malignancy	Normal ATOR. Hyperdense retrospectively.
17	59 years 7 months, F	Left-sided facial palsy and headache	Right frontal lobe	17 months	Present	Right parietal (multifocal)	MPRAGE	Not present	Reported as present	Present	Infarct, DDx malignancy	Hyperdense ATOR. Hyperdense retrospectively.
18	66 years 1 month, M	Seizure	Right parietal lobe	31 months	Present	Right thalamus	T1 SPACE FAT SAT	Present	Reported as present	Present	HGG with satellite lesion	Hypodense ATOR. Mixed hyper and hypodensity retrospectively.
19	45 years 5 months, M	Seizure	Left frontal lobe	24 months	Present	Left centrum semiovale posteriorly (multifocal)	TSE	Present, smudgy	Reported as not present	Present	Tumour, most likely oligodendroglioma	Hypodense ATOR. Hyperdense retrospectively.
							MPRAGE	Present				
20	55 years 11 months, M	Left-sided myoclonus	Right frontal – precentral gyrus	8 months	Present	Right anterior insula (multicentric)	MPRAGE	Present	Reported as present	Present	Subacute infarct, demyelination, encephalitis,	Hyperdense ATOR. Hyperdense retrospectively.
21	70 years 5 months, F	Difficulty walking and left lower leg weakness	Right superior parietal lobe	12 months	Present	None	MPRAGE	Present	Reported as not present	Present	CNS neoplasm, metastatic disease, subacute infarct	Normal ATOR. Normal retrospectively.
22	62 years 1 month, F	Right-sided headache, hypertension, and visual disturbances	Right occipital lobe	20 months	Present	Right medial temporal lobe (multicentric)	No contrast given	No contrast given	Reported as not present	Present	PRES, cerebritis, encephalitis	No comment ATOR. Hyperdense retrospectively.
23	49 years 7 months, F	Seizure	Right parietal	44 months	Present	Multiple adjacent lesions in the right parietal lobe (multicentric)	MPRAGE	Present	Reported as not present	Present	Post-seizure changes, PRES, cerebritis.	Normal ATOR. Normal retrospectively.
24	77 years 8 months, F	Seizure	Left anterior frontal lobe	3 months	Present	None	MPRAGE	Present	Reported as present	Present	Stroke	Hyperdense ATOR. Hyperdense retrospectively.
25	74 years 1 month, F	Aphasia and slurred speech	Left frontal lobe adjacent to Broca’s area	45 months	Present	None	MPRAGE	Present	Reported as present	Present	LGG, possible GBM.	No comment ATOR. Hyperdense retrospectively.
26	73 years 9 months, M	Seizure	Left temporal Lobe	7 months	Present	Pulvinar and other left temporal and frontal areas (multifocal)	No contrast given	No contrast given	Not reported	Present	LGG, encephalitis, post-ictal change	Loss of grey-white matter differentiation ATOR. Hyperdense retrospectively.
27	47 years 2 months, F	Right-sided paraesthesia (face, body, UL/LL, torso)	Left parietal postcentral gyrus	58 months	Present	Local satellite lesions (multifocal)	TSE	Present	Reported as present	Present	Cortical/subcortical neoplasm, ganglioma, abscess.	Did not have CT.
							MPRAGE	Present				
28	53 years 0 months, F	Seizure	Left temporal lobe	Alive (13 months)	Present	None	MPRAGE	Present	Reported as not present	Present	LGG, cortical dysplasia	Hyperdense ATOR. Hyperdense retrospectively.
29	67 years 7 months, M	Incidental finding	Right occipital lobe	18 months	Present	None	MPRAGE	Not present	Reported as present	Present	LGG	Did not have CT.
30	56 years 11 months, M	Expressive dysphasia	Left frontal superior gyrus (paramedian)	Alive (58 months)	Present	None	MPRAGE	Present	Not reported	Present	Neoplasm	Mixed hyper and hypodensity ATOR. Mixed hyper and hypodensity retrospectively.
31	59 years 9 months, F	Confusion, dysphasia, and right facial droop	Left posterior temporal lobe	Alive (9 months)	Present	None	TSE	Present	Reported as present	Present	LGG	Hypodensity ATOR. Mixed hyper and hypodensity retrospectively.
							MPRAGE	Present				
32	63 years 0 months, M	Seizure	Left posterior temporal lobe	4 months	Present	Multiple satellite lesions in the same lobe (multifocal)	TSE	Present	Reported as present	Present	LGG	Normal ATOR. Hyperdense retrospectively.
							MPRAGE	Present				
33	78 years 2 months, M	Dysphasia	Left posterior temporal	18 months	Present	None	TSE	Present	Not reported	Present	Infarct	Normal ATOR. Hyperdense retrospectively.
34	69 years 1 month, M	Left facial weakness and speech disturbance	Right frontal lobe	10 months	Present	None	No contrast given	No contrast given	Reported as present	Present	Ischaemic stroke	Normal ATOR. Mixed hyper and hypodense retrospectively.

Imaging trends

On T2WI and FLAIR sequences, all 34 patients (100%) had signal change in the cortical region, while 33 of 34 patients (97%) had increased signal change in the subcortical region. The regions with increased FLAIR and T2WI signal for all patients (100%) demonstrated restricted diffusion. However, the interpreting radiologist reported the presence of restricted diffusion in 21 patients (62%). No comment was made about restricted diffusion in five patients (15%), and eight patients (23%) had restricted diffusion incorrectly reported as absent during the initial interpretation of their MRI.

Of the 30 patients who had T1WI, we observed contrast enhancement in the brain parenchyma of 25 patients (83%), corresponding to the area demonstrating signal change on T2WI and FLAIR sequences. Five patients (17%) lacked enhancement on post-contrast T1WI. In our experience, the T1 TSE post-contrast sequence was more sensitive than the MPRAGE post-contrast sequence.

Of the 34 patients in our study, 32 patients (94%) had CT imaging prior to progressing to MRI imaging of the brain. Twenty-three patients (72%) had an area of hyperdensity on CT, correlating with the area of restricted diffusion on MRI. Similarly, one patient (3%) had an area of hypodensity, and five patients (16%) had mixed hypo- and hyperdensity correlating with the area of restricted diffusion on MRI. Three patients (9%) had normal CT imaging.

Initial imaging features were thought to be suggestive of a variety of pathologies, including low-grade glioma, infarct, inflammation, post-seizure changes, and posterior reversible encephalopathy syndrome. This was in keeping with prior literature.

**Figure 1:**
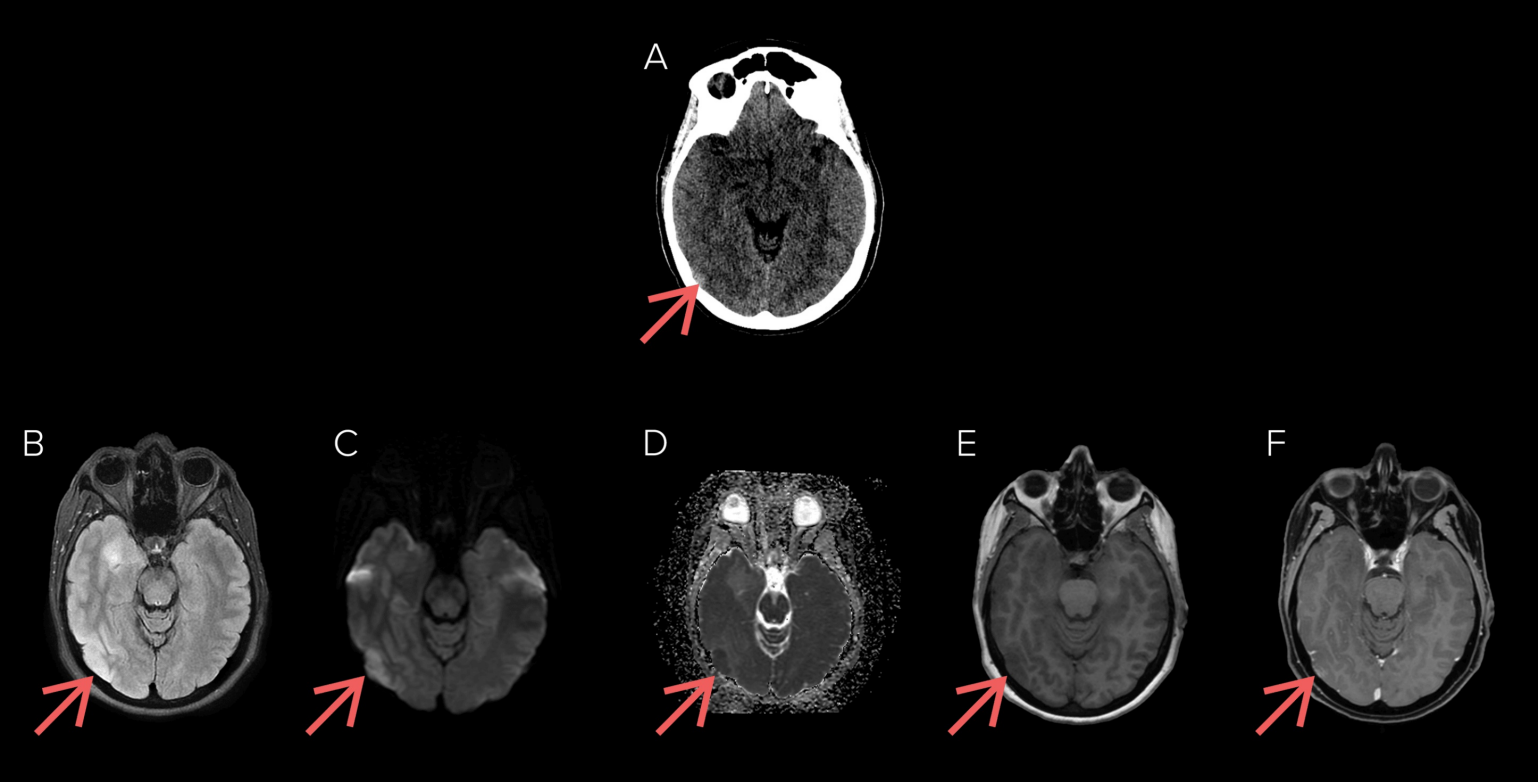
Computed tomography and gadolinium enhanced magnetic resonance images of the brain in a patient presenting with right-sided headache and visual disturbances. (a) Initial non-contrasted computed tomographic images revealed an area of hyperdensity in the right occipital lobe. (b) Fluid-attenuated inversion recovery (FLAIR), (c) diffusion-weighted imaging, (d) apparent diffusion coefficient, (e) pre-contrast T1, and (f) post-contrast T1 sequences revealed a poorly demarcated lesion corresponding to the area of hyperdensity seen on the initial computed tomography study, along with surrounding cerebral oedema. Note, in this case, that there is multicentric disease within the right mesial temporal lobe. Biopsy confirmed glioblastoma.

**Figure 2:**
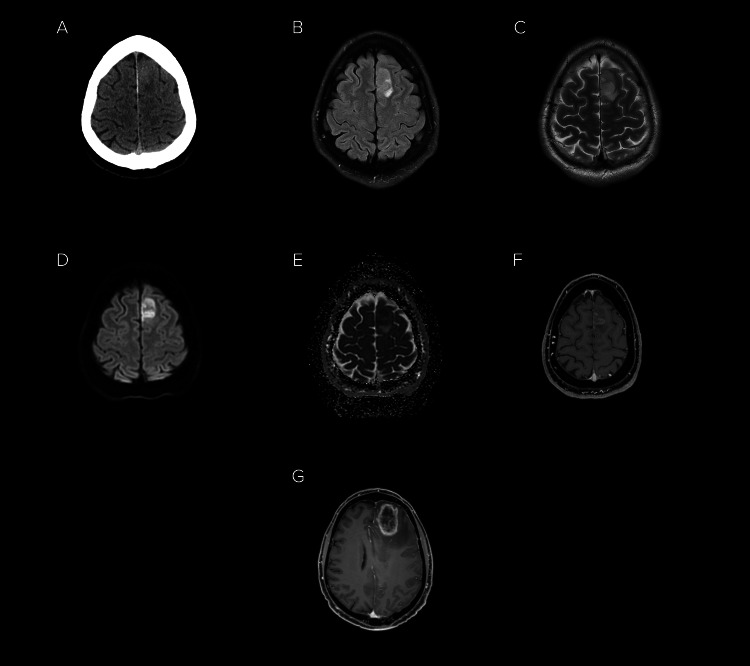
Computed tomography and gadolinium enhanced magnetic resonance imaging of the brain highlighting progression of early glioblastoma to classical appearance of glioblastoma. (a) Initial non-contrast computed tomography imaging in a patient presenting with seizure demonstrated a hyperdense region in the left superior frontal gyrus. Subsequent magnetic resonance imaging with (b) fluid-attenuated inversion recovery (FLAIR), (c) T2-weighted, (d) diffusion-weighted imaging, (e) apparent diffusion coefficient and (f) post-contrast T1 sequences showed an enhancing lesion with associated restricted diffusion. Biopsy confirmed glioblastoma. Despite treatment with surgical debulking and chemoradiotherapy, the patient had progression of disease, displaying (g) classic rim-enhancing lesion with irregular contrast enhancement, central necrosis and perilesional oedema on gadolinium-enhanced T1 imaging.

The imaging trends of e-GBM observed in our work are exemplified by Figure 1. Figure 2 outlines the evolution of e-GBM to classical GBM.

## Discussion

T2/FLAIR imaging findings

There is a consensus in the literature that e-GBM tends to present on MRI as small, T2-hyperintense lesions with no or subtle contrast enhancement [6,8,9,11-18]. In our patient cohort, we observed cortical T2-hyperintense lesions in all 34 patients and noted subcortical T2-hyperintense lesions in 33 of 34 patients, in keeping with previously published research findings. Additionally, all patients had FLAIR signal abnormalities at the location of T2-hyperintense lesions. Fourteen patients demonstrated T2 and FLAIR signal changes in the brain parenchyma remote from the index lesion. This pattern can mislead radiologists into discounting the possibility of an e-GBM, but we believe it represents multicentric or multifocal disease.

Diffusion weighted imaging

In addition to T2-hyperintensity, diffusion-weighted imaging is key in identifying e-GBM. This technique measures the Brownian motion of water molecules in tissues and is routinely performed in most modern clinical MR brain imaging protocols. It has been well documented that measured minimum apparent diffusion coefficient (ADC) values are inversely correlated with tumour cellularity and thus tumour grade, including for gliomas [19-21]. This is because higher-grade tumours are more cellular, with a smaller extracellular space, resulting in reduced diffusivity of water molecules.

We note that all patients in our series had a T2-hyperintense lesion that demonstrated restricted diffusion. One of the key challenges in imaging e-GBM is correctly identifying the presence of true restricted diffusion, this being a critical component in diagnosing e-GBM. As noted above, the presence of restricted diffusion was either not commented on or not correctly identified in approximately one quarter of our patients. Often, restricted diffusion may be subtle and therefore easily missed. Thus, we suggest that clinicians pay particular attention to the DWI and ADC sequences when reviewing imaging suspicious for e-GBM.

Similar results with DWI imaging of e-GBM were found by Wang and co-investigators [18]. In their series of eight pathologically diagnosed GBM, seven lesions demonstrated signal change on DWI. They were either heterogeneously hyperintense on DWI (n = 1) or isointense with a hyperintense region (n = 6). Thus, it is key that radiologists are vigilant for subtle areas of restricted diffusion in brain parenchyma with T2/FLAIR signal abnormalities. If seen, it is highly suspicious for e-GBM.

Contrast enhancement in magnetic resonance imaging

Contrast administration is of use when faced with potential cases of e-GBM. Enhancement in GBM occurs secondary to microvascular proliferation and disruption of the normal blood-brain barrier, with subsequent leakage of gadolinium into the extracellular interstitial space [22]. Indeed, contrast enhancement comprises part of the typical appearance of GBM on MRI, and it was suggested in older literature that the degree of enhancement was related to the degree of malignancy [23]. It has since been documented, however, that some cases of GBM are non-enhancing [6,24,25], and that some low-grade gliomas can also show contrast enhancement [26]. When present, contrast enhancement in e-GBM has been reported as focal and nodular [12,27,28].

In our work, when gadolinium was administered, contrast enhancement was seen in 25 patients, while five patients had no contrast enhancement. Enhancement patterns ranged from focal “smudging” of contrast to more prominent enhancement across areas of T2-hyperintensity. Ceravolo and collaborators noted in their work that several histologically proven e-GBM lesions were non-enhancing on contrast studies [11]. Thus, while contrast enhancement on T1 imaging may raise suspicion for e-GBM, it is important to note that not all e-GBMs display contrast enhancement.

In our experience, T1 TSE post-contrast sequences (including T1 SPACE) were more sensitive than T1 MPRAGE post-contrast sequences. Similar findings have been reported in the literature by Danieli and colleagues, who noted improved conspicuity in brain tumour enhancement using TSE techniques as opposed to MPRAGE [29]. This is important, as the latter sequence is a “workhorse” sequence in neurosurgical MRI. 

Computed tomography findings

Considered the workhorse of cross-sectional imaging, computed tomography is often employed as an initial investigation in neuroimaging. In our series, 32 of 34 patients had CT imaging of the brain undertaken prior to the index MRI. Of these 32 patients, 23 had an area of hyperdensity congruent with the location of their e-GBM on MRI (specifically the area of restricted diffusion), five had mixed hyper- and hypodensity, one had an area of hypodensity, and three were normal. Importantly, 13 of 32 CT reports did not identify the area of abnormal cerebral parenchyma.

Similar findings were noted by Wang and colleagues in their series of e-GBM patients. Of seven e-GBM patients with signal change on DWI sequences, six had a hyperdense region on CT and one had a hypodense region corresponding to the area of signal change on DWI. This led Wang and colleagues to conclude that CT and MRI with DWI sequences may be more accurate than routine MRI imaging alone in diagnosing e-GBM [18].

CT imaging in the setting of e-GBM was also examined by Ceravolo and colleagues in their series of 14 e-GBM patients. A total of eight patients had plain CT in conjunction with an MRI study of the brain. Of these patients, seven demonstrated regions of hyperdensity corresponding to the lesion detected on MRI [11]. Our work supports the position adopted by Ceravolo, Wang, and colleagues.

The importance of clinical history

In addition to imaging findings, having an accurate patient history is very important. All but one patient in our series presented with neurological findings, likely secondary to the cortical lesions. The most common clinical presentation was seizures, occurring in 18 of 34 patients. Similar findings have been reported by other authors [5,8].

In our experience, the most frequent diagnoses offered by reporting radiologists were low-grade glioma, followed by infarct, inflammation, and post-ictal change. For a diagnosis of infarct to be considered, there needs to be a history of the immediate onset of symptoms. It should also be noted that a seizure would be a very uncommon presenting symptom of an infarct.

Abnormal restricted diffusion can be seen in a few pathologies other than infarct, such as post-ictal change. Although there are some shared radiological characteristics [30], post-ictal changes should be transient and thus absent at follow-up imaging. An important exception is status epilepticus, which can cause permanent gliosis and focal atrophy, though the restricted diffusion itself usually resolves. Encephalitis is another example of a pathology that shows restricted diffusion, though the clinical history should help differentiate this from e-GBM.

Following up on suspicious lesions

We suggest short-interval progress imaging to monitor for progression and to differentiate from post-ictal change, especially in patients presenting with seizure. Based on previous reports of an approximately one-month volumetric doubling time of GBM [31], we suggest repeat imaging at three weeks. Early neurosurgical opinion should be sought, with a view to achieving a potential cure of these aggressive early tumours.

It is important to acknowledge the limitations of our study. Our work is retrospective due to the rapid progression exhibited by e-GBM. Furthermore, in keeping with other studies pertaining to e-GBM, our work is limited by a small sample size, owing to the rarity of early imaging in this cohort of patients. Finally, not all patients underwent the same sequence of imaging. Standardisation of MRI sequences is difficult to achieve, especially in the initial “discovery” MRI that first revealed an e-GBM in patients. In our series, these investigations were often undertaken at an external imaging site prior to referral to our service for ongoing management. Thus, prospective future work with a standardised MRI sequence would be invaluable in better understanding the imaging findings of e-GBM.

## Conclusions

We suggest that e-GBM should be better recognised and prioritised by radiologists as a differential diagnosis when there are T2WI or FLAIR signal abnormalities in a cortical or subcortical location, and if there is associated abnormal restricted diffusion, especially in the setting of corresponding hyperdensity on non-contrast CT of the head. Previous imaging can hold the key. Additionally, care should be taken to clarify the presenting symptoms at the time of imaging. The presence of focal neurological symptoms, such as a seizure, should further prompt e-GBM as a differential diagnosis. Several methodological improvements could add credence to our findings. Firstly, given that only one radiologist reviewed the imaging, increasing the number of reviewers and assessing inter-reviewer reliability could further validate the observations. Expanding the study to increase the sample size would also add weight to the validity of the findings. Additionally, using quantitative metrics, including volumetric measurements, ADC thresholds, perfusion metrics, or radiomics features, could allow more objective identification of e-GBM.

Future work may explore using artificial intelligence and machine learning in detecting subtle radiological features elusive to the human eye, especially with respect to the initial review of the preceding CT head. Pairing findings with other blood-based or CSF biomarkers, such as circulating tumour DNA and exosomes, may result in earlier and less invasive treatment. Examining GBM subtypes that have resulted in these appearances may also be of benefit.

## Appendices

**Table 3:** Search strings utilised for the literature review

Database searched	Search string
PubMed	Includes MeSH. ("Early Diagnosis"[Mesh] OR “early stage”[tiab] OR “early-stage”[tiab] OR “early”[tiab]) AND ("Glioblastoma"[Mesh] OR “glioblastoma”[tiab] OR “glioblastomas”[tiab]) AND ("Magnetic Resonance Imaging"[Mesh] OR “magnetic resonance imaging”[tiab] OR “MR imaging”[tiab] OR “MR images”[tiab] OR “MR image”[tiab] OR “MR scan”[tiab] OR “MR scans”[tiab] OR “MRI”[tiab] OR “MRIs”[tiab])
Embase	Includes Emtree; limited to articles, articles in press and reviews. ('early diagnosis'/exp OR “early stage”:ti,ab OR “early-stage”:ti,ab OR “early”:ti,ab) AND ('glioblastoma'/exp OR “glioblastoma”:ti,ab OR “glioblastomas”:ti,ab) AND ('nuclear magnetic resonance imaging'/exp OR “magnetic resonance imaging”:ti,ab OR “MR imaging”:ti,ab OR “MR images”:ti,ab OR “MR image”:ti,ab OR “MR scan”:ti,ab OR “MR scans”:ti,ab OR “MRI”:ti,ab OR “MRIs”:ti,ab) AND ([article]/lim OR [article in press]/lim OR [review]/lim)
SCOPUS	Advanced document search. TITLE-ABS ((“early stage” OR “early-stage” OR “early”) AND (“glioblastoma” OR “glioblastomas”) AND (“magnetic resonance imaging” OR “MR imaging” OR “MR images” OR “MR image” OR “MR scan” OR “MR scans” OR “MRI” OR “MRIs”))
